# Variation in Male Reproductive Longevity across Traditional Societies

**DOI:** 10.1371/journal.pone.0112236

**Published:** 2014-11-18

**Authors:** Lucio Vinicius, Ruth Mace, Andrea Migliano

**Affiliations:** Department of Anthropology, University College London, London, United Kingdom; University of Bern, Switzerland

## Abstract

Most accounts of human life history propose that women have short reproductive spans relative to their adult lifespans, while men not only remain fertile but carry on reproducing until late life. Here we argue that studies have overlooked evidence for variation in male reproductive ageing across human populations. We apply a Bayesian approach to census data from Agta hunter-gatherers and Gambian farmers to show that long post-reproductive lifespans characterise not only women but also males in some traditional human populations. We calculate three indices of reproductive ageing in men (oldest age at reproduction, male late-life reproduction, and post-reproductive representation) and identify a continuum of male reproductive longevity across eight traditional societies ranging from !Kung, Hadza and Agta hunter-gatherers exhibiting low levels of polygyny, early age at last reproduction and long post-reproductive lifespans, to male Gambian agriculturalists and Turkana pastoralists showing higher levels of polygyny, late-life reproduction and shorter post-reproductive lifespans. We conclude that the uniquely human detachment between rates of somatic senescence and reproductive decline, and the existence of post-reproductive lifespans, are features of both male and female life histories, and therefore not exclusive consequences of menopause.

## Introduction

Human adult life is characterised by slow senescence, mid-life fertility decline and a long post-reproductive lifespan [1]. Our extended post-reproductive life has been recently described as the result of a unique detachment between rates of reproductive and somatic senescence not found in other primates [2]. However, virtually all existing theories describe a long post-reproductive lifespan as an exclusive feature of women’s life history. The emphasis on women is justified by the universality of menopause, or full reproductive cessation and sterility typically occurring years or even decades before death [3]. Oldest ages at last reproduction (OLR) typically occur in women in their forties or fifties in hunter-gatherers, horticulturalists, pastoralists and farmers, as well as in historic and contemporary societies, with menopause and irreversible infertility taking place a few years later [4].

In contrast, most models postulate that as a rule men carry on reproducing into late life [5]. For example, Marlowe’s patriarch hypothesis [6], and its generalisation by Tuljapurkar et al. [7], proposes that male late-life reproduction drove the postponement of somatic senescence and lifespan extension in both sexes (with reproductive arrest occurring only in women due to oocyte depletion), and is therefore the explanation for human extended longevity. A rare exception to the characterisation of men as reproducing until late life was proposed by Kaplan et al. [8], who argued that intergenerational transfers, skill learning and late-life trade-offs would imply similar ages at last reproduction and long post-reproductive periods both in male and female Tsimane forager-horticulturalists.

The models above have in common the fact that they do not investigate the possibility that male reproductive lifespans may vary among human populations. Current evidence strongly suggests that, as a rule, men remain fertile in late life, with a few reports of age-related declines in spermatogenesis, but no evidence of widespread male infertility in mid- or late life [5]. The absence of an ‘andropause’ (or male equivalent to menopause) implies that men can potentially reproduce at old age; however, late-life male reproduction depends not only on fertility but on access to younger females, which depends on local levels of polygyny and serial monogamy known to differ significantly across traditional populations [9]. Unfortunately, most models of reproductive ageing in women and men, such as the Grandmother Hypothesis and the Patriarch Hypothesis, were based on data from a single population (in the two cases above, the Hadza hunter-gatherers) and do not involve comparisons across societies. Progress was made by Tuljapurkar et al. [7] whose tests were based on five traditional populations of hunter-gatherers, foragers and farmers. However, they also concluded that late-life reproduction is a typical feature of male life history, despite the fact that their measures of male realised fertility at old age clearly differed across the five societies.

Here we analyse evidence for differences in the duration of male post-reproductive lifespans in eight traditional populations, the largest sample assembled in a comparative study of reproductive senescence in men, and the first to include hunter-gatherers (!Kung, Hadza, Agta), forager-horticulturalists (Ache, Tsimane, Yanomamo), farmers (rural Gambians) and pastoralists (Turkana). We apply a new Bayesian methodology to analyse census data for two populations (Agta hunter-gatherers from the Philippines, and Gambian agropastoralists) and show that a detachment between somatic and reproductive senescence and long post-reproductive lifespans may characterise not only menopausal women [2] but also men. We reveal a continuum of male reproductive prolongation relative to women across populations, with !Kung, Hadza and Agta hunters exhibiting low levels of late-life polygyny, reproductive cessation almost as early as women, and long post-reproductive lifespans, and Turkana pastoralists and Gambian farmers showing an opposite pattern of late-life polygyny, late-life male reproduction and relatively shorter post-reproductive male lifespans. Together, our results demonstrate the existence of variation in late-life reproduction and post-reproductive lifespans in men.

## Methods

### Bayesian estimation of age-dependent survival and probability of last reproduction

Estimating survival trajectories for traditional populations is often problematic due to small samples and large number of right-censored cases (individuals still alive and thus not providing death date information) and hence indirect methods such as model life tables are frequently applied [10]–[11]. Here we apply a Bayesian methodology of demographic estimation [12] to census data (supplemented by interviews with information of deceased relatives) from the Agta from the Philippines and rural Gambians. Data on the Agta have been collected for almost fifty years [13], providing a sample of 1189 individuals still adopting a hunter-gatherer lifestyle (593 women and 596 men; 666 with a recorded death date and 523 still alive in 2008). Rural Gambians from three villages in the West Kiang district (Kantong Kunda, Keneba and Manduar) have been studied since 1950 by the UK Medical Research Council [14]. In 1974, the UK MRC established a clinic that significantly reduced child mortality rates in the villages. We selected a sample of 1667 individuals (788 women and 879 men; 411 with recorded death dates and 1256 still alive in 1997) born before 1975. To fit our data, we selected a Gompertz model that postulates mortality rates exponentially increasing with age [15]. When estimating the rate of ageing, we fit models to a subsample of individuals aged 15 years and over, thereby only modelling adult mortality (for that reason, our models show higher probabilities of survival at any age than survival curves starting from birth). The estimated Gompertz coefficient *b* represents the ‘rate of ageing’ or age-dependent increase in the probability of death [16].

**Figure 1:**
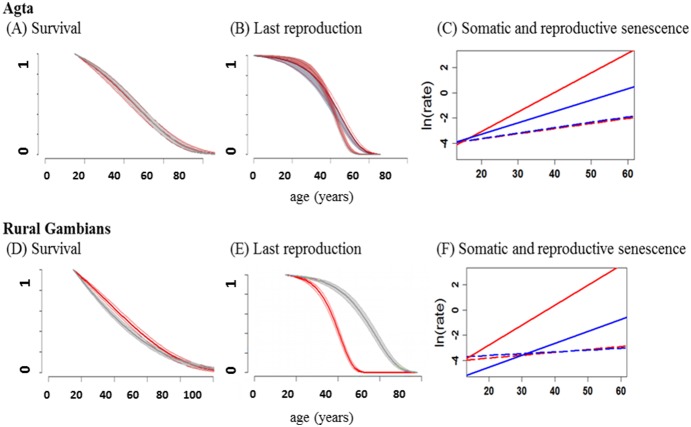
Survival and reproductive senescence curves for Agta and rural Gambians. In all panels, red lines represent female curves and blue lines represent male curves. (A) and (D): survival curves (probability of being alive by age) with 95% credible intervals. Curves estimate adult mortality only, and start with survival = 1 at age 15. (B) and (E): last reproduction curves (probability of not having reached last reproduction by age). Probability of last reproduction not having occurred until the age of around 15 is near 100%, when reproduction starts and it begins to decline. (C) and (F): regression lines of ln (mortality rate) by age (dashed lines) and ln (probability of reaching last reproduction) (solid lines). Dashed lines: regression slope is the rate of somatic senescence *b_som_*. Solid lines: regression slope is the rate of reproductive senescence *b_rep_*.

We adapted the Bayesian procedure to calculate probabilities of last reproduction by age. In this case, we first built a matrix (formally equivalent to a capture-recapture/recovery matrix) for each population and both sexes, with data on each row describing an individual reproductive history, and each column representing a year in which reproduction has occurred (denoted by 1) or not occurred (denoted by 0). We used this matrix to estimate Gompertzian curves of age-dependent probability of last reproduction; the curves are therefore formally equivalent to mortality curves representing age-dependent probability of death. Since models are now based on reproduction data, the estimated Gompertz coefficient *b* represents the ‘rate of reproductive senescence’, or age-dependent increase in the probability of last reproduction (‘reproductive death’). Our definition of reproductive senescence therefore relates exclusively to the event of reproductive cessation in men and women, and not to any underlying physiological process (such as menopause in women). Based on the two coefficients *b_som_* (rate of ageing or somatic senescence) and *b_rep_* (rate of reproductive senescence), we extend the comparison between somatic vs. reproductive senescence in !Kung women proposed by Alberts et al. [2] to male and female Agta and rural Gambians. In order to visually compare rates of ageing and reproductive senescence, we linearised our Gompertz curves and present them as regression lines in Figure 1. All Bayesian procedures were implemented using the *R* package *BaSTA* [17]. Details of the mathematical model and simulations are presented in File S1.

### Oldest age at last reproduction (OLR), age-dependent survival and fertility, and polygyny

Data on oldest recorded age at last reproduction (OLR), age-dependent survival and fertility, and polygyny (meaning the fraction of married men having more than one wife) in the !Kung and Ache are respectively from Howell [18] and Hill and Hurtado [19]. Hadza OLR, age-specific survival and fertility are from Blurton-Jones [20] and polygyny rate from Marlowe [21]. OLR and age-dependent fertility in the Agta and rural Gambians are from the datasets described above, survival curves were estimated though our Bayesian procedure described above, and polygyny figures are respectively from Early and Headland [22] and Ratcliffe et al. [23]. Tsimane and Yanomamo OLR and fertility curves are from Tuljapurkar et al. [7], survival curves respectively from Gurven, Kaplan and Zelada Supa [24] and Neel and Weiss [25], and polygyny data respectively from Winking et al. [26] and Early and Peters [27]. All Turkana data are from Little and Leslie [28].

### Male late-life reproduction (MLR)

Tuljapurkar et al. [7] calculated the contribution of late life to reproduction in men as the ratio of realised male fertility after the age of menopause divided by total realised male fertility. However, this measure does not take into account the impact of decreasing fertility in late life, i.e. it does not weigh age-specific fertility (*m_x_*) by age-specific mortality (*l_x_*). Sums or integrals of age-dependent *l_x_m_x_* products are used in measures of fitness such as the Euler-Lotka equation or the net reproductive rate 
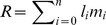
 [29]. For this reason, we defined male late-life fertility (MLR) as the net reproductive rate of men after the age of last female reproduction (male *R* after the age at last female reproduction) divided by total net reproductive rate (total male *R*). We also estimated post-reproductive representation (PrR), a measure of the post-reproductive fraction of adult life, based on survival and fertility curves from the eight populations and both sexes. For more details on definition and calculation of PrR see ref [1]. To calculate life expectancies from birth and PrR for Agta and rural Gambians, we fit Siler (U-shaped) mortality curves to datasets (using data from birth) using the software *BaSTA* and the Bayesian procedures described above and in File S1, and then estimated the appropriate integrals from the obtained survival curves.

## Results

### Somatic vs. reproductive senescence: Agta and rural Gambians

We compared rates of somatic and reproductive senescence in two populations using Gompertz models of age-dependent survival and probability of last reproduction (Figure 1). In Agta women, the rate of increase in the probability of last reproduction (*b_rep_* = 0.155, 95% credible interval = 0.135–0.178, DIC = 13573.6) is significantly higher than the Gompertz rate of ageing (*b_som_* = 0.041, 95% credible interval = 0.033–0.049, DIC = 11996.34). The same is true for Gambian women (*b_rep_* = 0.158, 95% credible interval = 0.141–0.178, DIC = 8095.3; *b_som_* = 0.023, 95% credible interval = 0.018–0.028, DIC = 19879.72). This is expected in women due to menopause, however differences between rates of somatic and reproductive senescence were also observed in men, both in the Agta (*b_rep_* = 0.09, 95% credible interval = 0.078–0.102, DIC = 13573.6; *b_som_* = 0.043; 95% credible interval, 0.036–0.051, DIC = 11996.34) and rural Gambians (*b_rep_* = 0.095, 95% credible interval = 0.085–0.105, DIC = 8095.3; *b_som_* = 0.014, 95% credible interval = 0.010–0.019, DIC = 19879.72). Therefore the detachment between rates of ageing and reproductive senescence, which differentiates humans from other primates, is not an exclusive consequence of menopause and is observed in men from two populations markedly differing in adult survival [30]–[31].

### Variation in male late-life reproduction

We also found significant evidence for variation in ages of reproductive termination in men across traditional populations. Male OLR reported in the Dobe !Kung was 54 years old [18] while in rural Gambians male reproduction extended until the age of 78. The eight populations reveal male OLR in the mid-fifties in hunter-gathering !Kung and Hadza, early and mid-sixties in Tsimane and Yanomamo horticulturalists and Agta hunter-gatherers, and seventies in Turkana pastoralists and Gambian agropastoralists (Table 1). There is one case of male OLR between the ages of 65 and 75 in the Ache forager-horticulturalists [19], but individual age was not reported. In summary, while the maximum recorded age at last reproduction in women spans over 13 years (45–58 years) in the eight traditional populations, the span is over two decades in man (54–78 years).

**Table 1:** Reproduction, economy and polygyny in eight traditional populations.

Group	Economy	OLR		MLR (%)	PrR			Polygyny (%)
		men	women		men	women	ratio	
!Kung	Hunter-gatherer	54	46	3.6	0.460	0.505	0.91	6.3
Hadza	Hunter-gatherer	55	45	13.8	0.294	0.476	0.62	4
Agta	Hunter-gatherer	66	54	0.1	0.256	0.335	0.76	5
Ache	Forager-horticulturalist	65–75	48	12.9	0.135	0.417	0.32	4.1
Tsimane	Horticulturalist	60–64	45–49	7.1	0.185	0.354	0.53	5.8
Yanomamo	Horticulturalist	60–64	45–49	10.2	0.08	0.256	0.31	29.8
Turkana	Pastoralist	70+	51	51.1	0.006	0.395	0.015	79.8
Gambia	Agro-pastoralist	78	58	29.9	0.200	0.462	0.43	40

OLR: oldest recorded age at last reproduction by a mother or a father. For the Ache, Tsimane, Yanomamo and Turkana, individual data were not available and we therefore used age intervals. MLR: male late-life reproduction, or fraction of net reproductive rate in men realised after the age at last reproduction in women. PrR: post-reproductive representation, or post-reproductive fraction of adult life, in men, women, and ratio of male to female PrR. Polygyny is the percentage of males with more than one wife at the time of data collection. See Methods for data sources.

Like maximum observed longevity, OLR is sensitive to factors such as small sample size or a single exceptional individual [1], and we therefore calculated other measures of reproductive longevity in men. MLR (male-late life reproduction), which we defined as the fraction of male net reproductive rate realised after the age of female last reproduction, also varies across sampled populations. MLR values (which depend on sums of *l_x_m_x_* products, or age-dependent fertility *m_x_* weighed by age-dependent survival *l_x_*) are significantly smaller than the fraction of male late-life realised fertility (based only on *m_x_*) calculated by Tuljapurkar et al. [7]. MLR is only 3.6% in the Dobe !Kung (compared to 4.9% of late-life realised fertility), 12.9% in the Ache (19.3% realised fertility), 10.2% in the Yanomamo (20.6% realised fertility), 7.2% in the Tsimane (9.9% realised fertility), and 29.9% in rural Gambians (36.4% realised fertility). We also estimated male late-life reproductive rate in the Hadza (13.8%) and Agta hunter-gatherers (0.1%), and Turkana pastoralists (51.1%). The Agta value was based on an oldest age of female reproduction of 58 years, but even if this outlier is neglected and the age at termination of female reproduction is set at 50 years, the contribution of late life to male net reproductive rate would be only 0.7%. Therefore, only in rural Gambians and Turkana pastoralists do men realise over 20% of their net reproductive rate after women have had their last child. Contrary to widespread views, there is significant variation across populations in the duration of male reproductive spans.

### Distribution of male post-reproductive lifespans

The detachment between somatic and reproductive senescence in both sexes and variation in contribution of late-life reproduction suggests differences in the duration of male post-reproductive lifespans across populations. Levitis et al. [1] have shown that post-reproductive representation (PrR), or the ratio of post-reproductive lifespan to total reproductive lifespan, is significantly longer in human populations than in other primates, but their analyses only included females. We calculated post-reproductive lifespans in both men and women (Table 1). Female PrR ranges from 0.256 (Yanonamo) to 0.51 (!Kung), while in men values ranged from 0.006 in the Turkana to 0.46 in the !Kung. In relative terms, male post-reproductive spans vary from only 1.5% of the female total in the Turkana, to 75% in the Agta and 91% in the !Kung.

**Figure 2:**
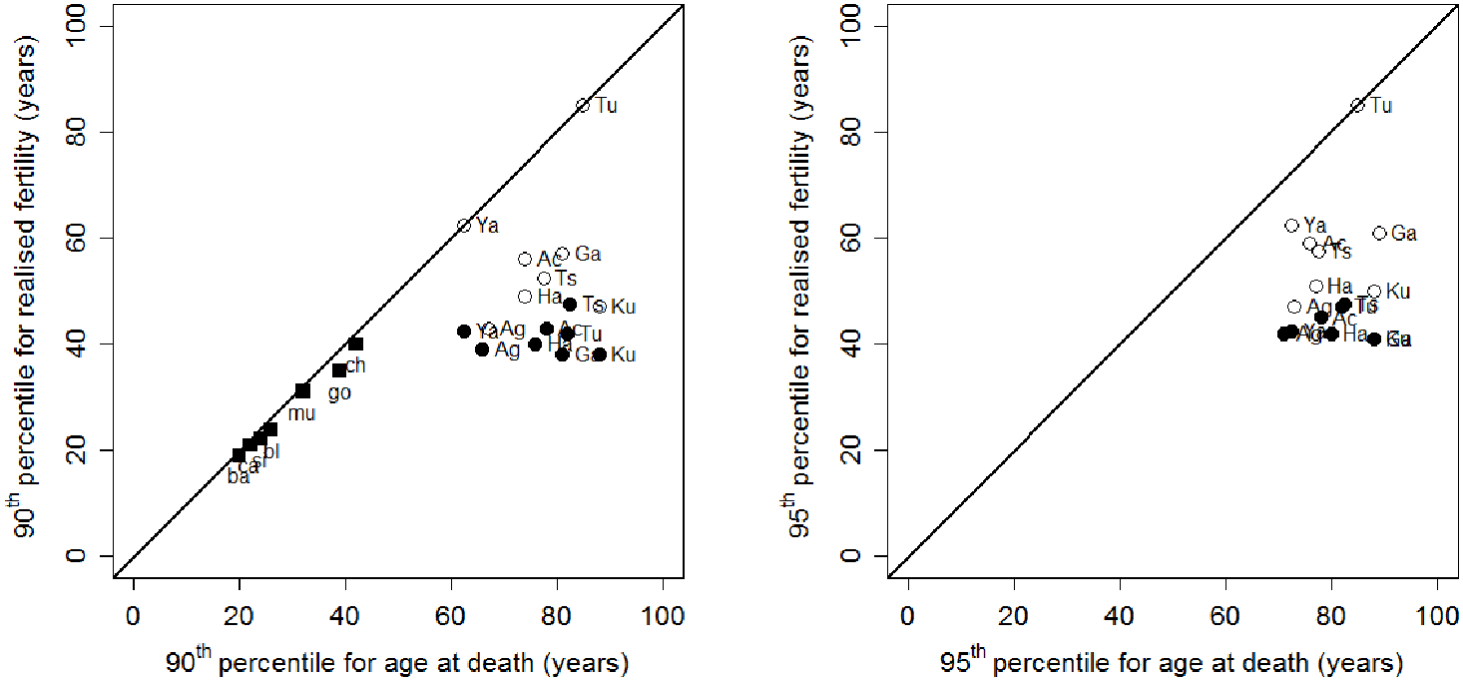
90^th^ and 95^th^ percentiles of realised fertility vs. realised survival in human populations, in years. Left panel: 90^th^ percentile in humans and non-human primates. Solid squares: 90^th^ percentiles in primate species. Data on baboon (ba), capuchins (ca), sifaka (si), blue monkey (bl), muriqui (mu), gorilla (go) and chimpanzee (ch) are from Alberts et al. (2013). Human data (95^th^ percentiles): Ache (Ac), Agta (Ag), Hadza (Ha), !Kung (Ku), Tsimane (Ts), Turkana (Tu) and Yanomamo (Ya). Data sources: see methods. Open circles: men. Solid circles: women. Primate species, plus Turkana and Yanomamo males, are close to the bisecting line *y* = *x*, indicating similar rates of somatic and reproductive senescence. For the other male populations and all females, the 90^th^ percentile of realised survival is reached at an older age than realised fertility, indicating a faster rate of reproductive than somatic senescence. Right panel: 95^th^ percentile in human populations. Legends as above. In all male populations, the 95^th^ percentile of realised survival is reached at an older age than realised fertility.

Alberts et al. [2] plotted ages at 90% of realised fertility (age at which 90% of the reproductive output has been completed) against ages at 90% of realised survival (age at which 90% of the people in the population have died) for seven female primates and !Kung women, and showed that only in humans did the two ages significantly differ. We extended their analysis to both sexes and eight human populations. Figure 2 shows that the detachment between ages at 90% of realised survival and reproduction is a characteristic of women in all eight populations, and also typical of all male populations with the exception of the Turkana and Yanomamo. In addition, age at 95% of realised reproduction is lower than age at 95% of realised survival in all human populations except the Turkana (Figure 2). The results show that long post-reproductive lifespans are a feature of male life histories in some populations, while in others male reproduction extends until near the end of life.

### Variation in male late reproduction and polygyny levels

**Figure 3:**
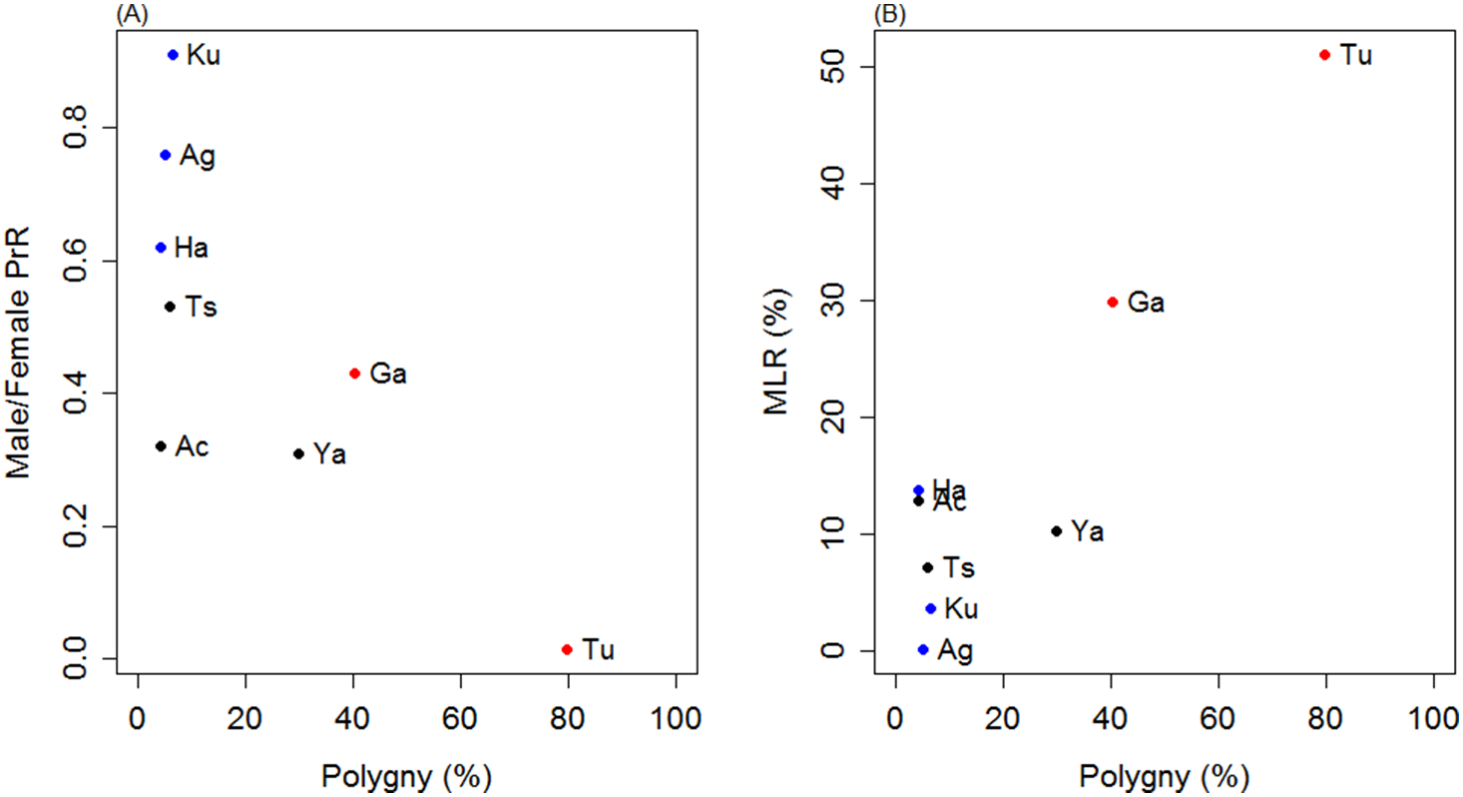
Male reproduction in relation to polygyny and economic basis. (A) Ratio of male to female PrR (post-reproductive representation) vs. polygyny. (B) MLR (male late-life reproduction) vs. polygyny. Legends as in Figure 2. Hunter-gatherers in blue, forager-horticulturalists in black, and pastoralists and agropastoralists in red.

We also compiled data on polygyny levels in the eight populations (Table 1, Figure 3). The ratio of male to female post-reproductive representation (male PrR divided by female PrR) is lowest in the Turkana pastoralists (0.015), where polygyny is the highest amongst the eight populations (79.8%). At the other extreme, the three hunter-gatherer groups (Dobe !Kung, Agta and Hadza) show the highest PrR ratios (0.91, 0.76 and 0.62) and lowest polygyny levels (6.3%, 5%, 4%).

The Dobe !Kung, Hadza, Agta, Ache and Tsimane also show the lowest levels of MLR (13.8% or less), and polygyny levels below 10%. Yanomamo horticulturalists show high levels of polygyny (29.8%) and low levels of MLR (10.2%). High levels of MLR are observed in rural Gambians (29.9%) and Turkana pastoralists (51%), where polygyny is widespread at 40.2% and 79.8% respectively.

## Discussion

We have shown that late-life reproduction and duration of post-reproductive lifespans in men are highly variable across traditional populations. Previous claims that men either reproduce until much later than women [6]–[7] or stop reproduction almost at the same time as women [8] seem to be based on extreme cases such as rural Gambians or the Agta. Data compiled from eight traditional populations suggest instead the existence of a continuum of male late-life reproduction, with hunter-gathering Agta and Dobe !Kung men stopping reproduction nearly as early as women, while male Turkana pastoralists and rural Gambian farmers significantly extend reproduction into late life. The three forager-horticulturalists in our sample (Ache, Tsimane and Yanomamo) displayed intermediate values of male late-life reproduction. Our results therefore contradict the claims of widespread reproduction at old age in men proposed by Tuljapurkar et al. [7], who relied on measures of realised fertility that overestimate the contribution of late-life to male reproduction.

Between-population variation in the timing of male reproductive cessation contrasts with the more rigid pattern of reproductive senescence in women. While menopause occurs universally and prevents reproduction in late life, studies of male reproductive physiology have reported only ‘modest’ changes in spermatogenesis at old age, with possible effects on fertility remaining questionable [32]. Since there is no evidence of widespread male mid- or late-life sterility, variation in late-life reproduction in men must therefore reflect differences in opportunities to reproduce at old age. Among the eight populations in our study, the Turkana pastoralists exhibit the highest levels of male late-life reproduction and shortest post-reproductive representation, and the high levels of polygyny in this population may be providing access of older men to younger women at reproductive age. At the other extreme, the hunter-gathering !Kung and Agta showed lowest levels of male late-life reproduction and low levels of polygyny. It is interesting to notice that the two populations showing the highest levels of male late-life reproduction are the Turkana pastoralists and Gambian farmers, which suggests that the occurrence of late-reproducing males may be a recent event in human societies, possibly as a result of changes in food production systems. However, a larger sample of traditional populations is required before a more general assessment of relations between male reproductive spans, polygyny levels and subsistence modes can be established.

Finally, our results are relevant for evolutionary accounts of human life history. Alberts et al. [2] have shown that long post-reproductive lifespans are a unique human feature. However, their evidence is exclusively derived from curves of probability of last reproduction in women, which suggests an association between long post-reproductive lifespans and the occurrence of menopause. We have shown that post-reproductive lifespans are a feature of male life histories too, although their duration varies across populations. This conclusion raises important questions, such as determining how old the pattern of between-population variability in male late-life reproduction is, and defining possible adaptive reasons (if any) for it. In addition, variability in levels of late-life reproduction pose a direct challenge to the view that late-reproducing men are a general explanation for why humans live past the Hamiltonian ‘Wall of Death’ (the age at menopause) [7].

In summary, our study draws attention to the generally neglected topic of post-reproductive lifespans in men, which are significantly long in some traditional societies even in the absence of a post-fertile stage similar to observed in menopausal women. It also highlights a spectrum of variation in the duration of male reproductive spans across societies differing in polygyny levels and subsistence mode. Our sample of eight traditional populations is the largest so far assembled in a study of male reproductive senescence, and we hope that our study inspires collection and publication of data on male age-dependent reproduction in other traditional populations. More information, especially on pastoralist and farming societies, is needed to allow a proper statistical assessment of relationships between subsistence mode, polygyny and measures of reproductive senescence in men. In particular, data on Australian hunter-gatherers, ‘complex’ hunter-gatherers, and other pastoralist societies, presently unavailable or non-existent, could significantly extend our knowledge of variability and evolution of late-life reproduction, reproductive senescence and post-reproductive lifespans in men.

## Supporting Information

File S1 Additional information on Bayesian estimation of survival and probability of last reproduction curves.
(DOCX)
