# Supplementary material for: Variation in Male Reproductive Longevity across Traditional Societies
Source: PLoS One. 2014 Nov 18;9(11):e112236. doi: 10.1371/journal.pone.0112236 (PMC4236073; doi:10.1371/journal.pone.0112236)
Supplement: File S1 — Additional information on Bayesian estimation of survival and probability of last reproduction curves. (DOCX) [file pone.0112236.s001.docx]

Vinicius et. al. Variation in male reproductive longevity across traditional societies.

**Supplementary Information File S1.** Additional information on Bayesian estimation of survival and probability of last reproduction curves.

**Bayesian modelling of age-specific survival and fertility cessation.**

We have calculated age-dependent survival and fertility cessation curves in two populations (Agta and rural Gambians) using a Bayesian framework implemented by the *R* package *BaSTA* (Bayesian Survival Trajectory Analysis) [1-2]. *BaSTA* implements a Bayesian hierarchical model originally designed to analyse capture-recapture and capture-recovery data including both left-truncated (individuals with no known birth dates) and right-censored data (individuals still alive at the end of the study), which we applied to census data from Agta and rural Gambians characterised by a large fraction of right-censored cases, but no left-truncated individuals (as birth dates are either known of estimated from interviews). We recommend reading of the original articles by Colchero et al [1,2] with the full description of the model and package. Below we present a short summary (strongly based on refs 1-2) of their mathematical model and *BaSTA*.

*BaSTA* fits different parametric models to birth and death datasets. Hazard rates are generally defined as

$$\mu\left( x | \theta\right)=\lim_{dx\to0} \frac{\Pr\left( x\leq X<\left( x+\Delta x \right) | x\leq X,\theta\right)}{dx}$$

From this we obtain the survival function

$$S\left( x | \theta\right)=\Pr\left( X\geq x \right)=e^{-\int_{0}^{x} \mu\left( z | \theta\right)dz}$$

with *x*=age, *X*=age at death, and *θ*=model parameters. Hazard rates and survival functions take specific shapes depending on the choice of mortality model (Gompertz, Logistic, Siler etc.). The *BaSTA* algorithm is based on an approach that splits the posterior distribution of unknowns into three parts: estimation of survival parameters *θ*, estimation of unknown death ages, and estimation of probabilities of recapture. Since we are using census data, we are mostly interested in curve fitting or estimation of survival parameters of a chosen mortality model. The Bayesian hierarchical approach requires only the conditionals for posterior simulation by a Markov Chain Monte Carlo algorithm (Metropolis-within-Gibbs sampling). The density of the parameters *θ* conditioned on known and unknown death ages *X* is given by

*p*(*θ*|*X_k_*, *X_u_*) $\propto$ *p*(*X_u_*, *X_k_*|*θ)p*(*θ| θ_p_*)

where *θ_p_* are the parameter priors*.* The acceptance probability the potential vectors of parameters *θ and θ’* conditioned on real and proposed death ages is obtained via Metropolis sampling as

$$p\left( \theta, \theta^{'} \right)=min\left\{ 1, \frac{\prod_{1}^{n} [f\left( X_{i} | \theta^{'} \right]p(\theta^{'}|\theta_{p})}{\prod_{1}^{n} [f\left( X_{i} | \theta\right]p(\theta|\theta_{p})} \right\}$$

Converged sequences of parameter estimates *θ,* their derived means, and 95% credible intervals are the model outputs. Convergence is achieved when $\hat{R}=\sqrt{\frac{\hat{v}^{+}}{W}}$ < 1.1, where *W* is within-sequence variance and $\hat{v}^{+}$ is a weighted average of *W* and between-sequence variance. Model selection is based on DIC (deviance information criterion), which consists of a measure of goodness-of-fit and a penalisation for model complexity and is recommended when posterior distributions are obtained by MCMC algorithms.

**Simulation parameters and argument settings in *BaSTA***

We ran both Gompertz and Siler mortality models with the function *basta*(). We selected Gompertz models when estimating mortality and survival rates from age 15 years. The Gompertz model postulates that hazard rates are given by *μ*(*x*)= $e^{b_{0}+b_{1}x}$ (with $e^{b_{0}}$=*α*=baseline mortality and *b_1_*=rate of ageing, or rate of increase in probability of last reproduction in our models of age at last reproduction). Survival probability is then

*S*(*x*|*b_0_*, *b_1_*) = $e^{\frac{e^{b_{0}}}{b_{1}}(1-e^{b_{1}x})}$

The Siler model was applied to U-shape mortality patterns from birth. Its hazard rate is given by *μ*(*x*)= $e^{a_{0}-a_{1}x}+c+ e^{b_{0}-b_{1}x}$, and the corresponding survival probability is

*S*(*x*|*a_0_*, *a_1_*, *b_0_*, *b_1_*, *c*) = $e^{\left[ \frac{e^{a_{0}}}{a_{1}}\left( e^{-a_{1}x}-1 \right)+cx+\frac{e^{b_{0}}}{b_{1}}\left( {1-e}^{b_{1}x} \right) \right]}$

To fit Gompertz mortality curves, we add the argument *model= ‘GO’* to the function *basta*(). Selecting a Siler model requires the arguments *model= ‘GO’* and *shape= ‘bathtub’*. The argument *minAge=15* limits the procedures to ages over 15 years. The default value is *minAge=0*, which produces mortality and survival curves from birth. The number of iterations was set at between *niter=10000* and *niter=50000*, depending on the speed of parameter convergence. The burn-in sequences, or the number of pre-convergence and discarded initial steps, was set at the default value of *burnin=5001*. The thinning interval between consecutive parameter estimations was set at the default value of *thinning=50*. Priors have default values in *BaSTA*. Gompertz models have priors set at *b_0_*=-3.00 and *b_1_*=0.01, and Siler models have priors *a_0_*=-2.00, *b_0_*=-3.00, *a_1_*=*b_1_*=0.01 and *c*=0.

Supplementary File 2 (*R* code and workspace) includes all input files and *R* code, and most output files (which can all be produced by re-running simulations using the code provided). Notice that running simulations again will produce slightly different outcomes from those reported in the main article.

**References**

1. Colchero F, Clark JS (2012) Bayesian inference on age-specific survival for censored and truncated data. *J Anim Ecol* 81: 139-149.

2. Colchero F, Jones OR, Rebke M (2012) BaSTA: an R package for Bayesian estimation of age-specific survival from incomplete mark-recapture/recovery data with covariates. *Methods Ecol Evol* 3: 466-470.
